# A Gene Expression Signature of Invasive Potential in Metastatic Melanoma Cells

**DOI:** 10.1371/journal.pone.0008461

**Published:** 2009-12-24

**Authors:** Aaron R. Jeffs, Amy C. Glover, Lynn J. Slobbe, Li Wang, Shujie He, Jody A. Hazlett, Anshul Awasthi, Adele G. Woolley, Elaine S. Marshall, Wayne R. Joseph, Cristin G. Print, Bruce C. Baguley, Michael R. Eccles

**Affiliations:** 1 Department of Pathology, Dunedin School of Medicine, University of Otago, Dunedin, New Zealand; 2 Department of Biology, University of York, York, United Kingdom; 3 Department of Molecular Medicine and Pathology, School of Medical Sciences, University of Auckland, Auckland, New Zealand; 4 National School of Pharmacy, University of Otago, Dunedin, New Zealand; 5 Auckland Cancer Society Research Centre, University of Auckland, Auckland, New Zealand; Technical University Munich, Germany

## Abstract

**Background:**

We are investigating the molecular basis of melanoma by defining genomic characteristics that correlate with tumour phenotype in a novel panel of metastatic melanoma cell lines. The aim of this study is to identify new prognostic markers and therapeutic targets that might aid clinical cancer diagnosis and management.

**Principal Findings:**

Global transcript profiling identified a signature featuring decreased expression of developmental and lineage specification genes including *MITF*, *EDNRB*, *DCT*, and *TYR*, and increased expression of genes involved in interaction with the extracellular environment, such as *PLAUR*, *VCAN*, and *HIF1a*. Migration assays showed that the gene signature correlated with the invasive potential of the cell lines, and external validation by using publicly available data indicated that tumours with the invasive gene signature were less melanocytic and may be more aggressive. The invasion signature could be detected in both primary and metastatic tumours suggesting that gene expression conferring increased invasive potential in melanoma may occur independently of tumour stage.

**Conclusions:**

Our data supports the hypothesis that differential developmental gene expression may drive invasive potential in metastatic melanoma, and that melanoma heterogeneity may be explained by the differing capacity of melanoma cells to both withstand decreased expression of lineage specification genes and to respond to the tumour microenvironment. The invasion signature may provide new possibilities for predicting which primary tumours are more likely to metastasize, and which metastatic tumours might show a more aggressive clinical course.

## Introduction

Melanoma can progress rapidly from a slow-growing surgically curable lesion to aggressive metastatic disease, with high mortality and poor response to current therapies [1], but the mechanisms underlying melanoma progression and resistance to therapeutic agents are not well understood. There are few treatment options for melanoma once it has metastasized, and new biomarkers that aid diagnosis, predict clinical outcome, and suggest new therapies are required (reviewed in [2]). Surgical, chemical, and biological therapies offered to patients with metastatic melanoma are all essentially palliative in nature, with no way of predicting which patients will benefit. Metastatic melanoma is clinically heterogeneous [3], [4], with 5-year survival rates of less than 10% for those patients presenting with disseminated disease [5]. The best chance of surviving melanoma remains early detection and surgical resection of the primary tumour.

Melanoma incidence is reported to be increasing globally, with rates in New Zealand amongst the highest in the world. The lifetime risk of developing melanoma in fair-skinned New Zealanders is about 1 in 17 [6]. In 2004, melanoma was the third most common cancer registration in New Zealand females and fourth for males, and ranked ninth and sixth respectively in terms of cancer-related deaths [6]. About 2000 new cases occur annually in New Zealand with about 250 deaths, and although 50% of melanoma occurs in those aged over 60, melanoma is the leading cause of cancer deaths in 15–44 year old New Zealand males [6] resulting in loss of productive life years. The number of deaths caused by metastatic melanoma is unlikely to decrease in New Zealand in the near future as strategies to encourage the early detection of melanoma in New Zealand have not yet resulted in declining incidence of poorer prognosis thick primary melanomas [7].

Global gene expression profiling by using microarrays has proven to be a significant tool in helping to uncover the molecular basis of cancer. Molecular classification of different cancers, such as colorectal and lymphoma, has consistently stratified tumours into sub-types with prognostic outcomes independent of those suggested by conventional clinical staging procedures [8], [9]. Gene expression profiling of breast tumours has resulted in sub-classification of cancers previously thought to be homogenous [10], allowing prediction of those most likely to benefit from chemotherapy [11] and overall survival [12]. Gene expression profiling has generated a number of insights into the molecular basis of melanoma over the last decade ([13]-[16]; reviewed in [17]), but this accumulation of knowledge has yet to provide clinical benefit in terms of improved patient treatment options or survival.

We are investigating the molecular basis of melanoma biology and heterogeneity by characterising a novel panel of cell lines developed largely from New Zealand patients with metastatic melanoma. Global gene expression analysis showed that the cell lines could be stratified by differential expression of genes related to melanocyte development and differentiation, and that lower expression of MITF and related transcriptional networks combined with higher expression of environmental interaction genes correlated with increased invasive potential *in vitro*. Validation on independent cell line and tumour data suggests that our gene expression profile is a general invasion signature in melanoma associated with metastatic potential, and may occur independently of stage.

## Results

### Global Transcript Profiling Classified Cell Lines According to Differential Expression of Developmental and Tissue Remodelling Genes

**Figure 1:**
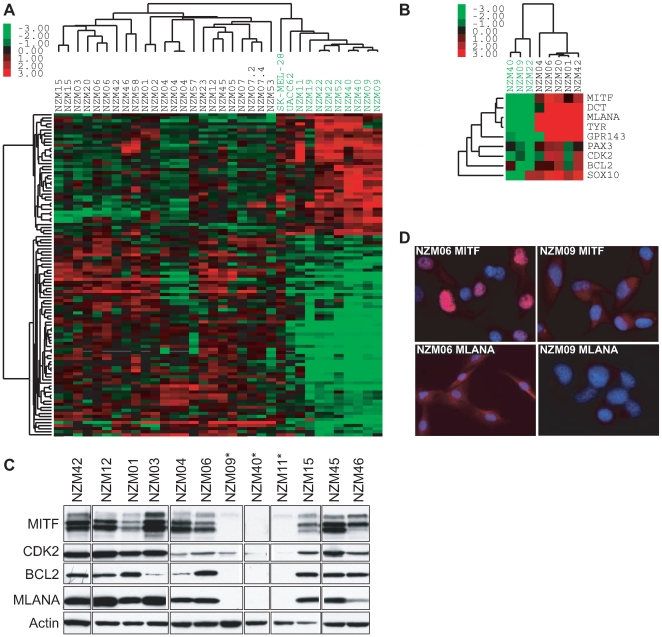
Expression profiling and validation. (A) Unsupervised hierarchical clustering by using 96 genes identified by class comparison as differentially expressed between relatively higher or lower *MITF*-expressing melanoma cell lines classified the cell lines into two main groups (Motif 1, green label; Motif 2, black label). (B) qPCR validation of selected targets in a subset of Motif 1 and 2 NZM cell lines. Unsupervised clustering of qPCR data confirmed the Motif 1 (green) and 2 (black) classifications. (C) Protein expression of MITF targets CDK2, BCL2, and MLANA agreed with *MITF* transcript levels and array and qPCR cell line stratification. *Motif 1 cell lines. (D) Immunofluorescence showed stronger staining for MITF and MLANA in the Motif 2 NZM06 cells compared to Motif 1 NZM09 cells.

After stringent microarray data filtering (Supplementary Information S1), 572 of the initial ∼20,000 transcripts on the array remained as input for further analysis (Supplementary Information S1). Unsupervised hierarchical clustering of the filtered gene list showed two main groups of cell lines (Figure S1). One of the gene clusters that distinguished the two main groups showed differential expression of neural crest markers such as *MITF* and other genes related to melanocyte development, differentiation, and function (Figure S1). Given the central role of MITF in melanocyte development, and the implication of MITF in melanoma progression, we investigated the consequences of differential MITF expression in more detail. Class comparison generated a list of 106 transcripts representing 96 unique genes that significantly discriminated between higher and lower MITF cell lines (p<0.001, FDR<1%; Table S1 and Supplementary Information S1). Hierarchical clustering using only the 106 transcripts identified by class comparison revealed two main expression motifs in the melanoma cell lines (Figure 1A): Motif 1 distinguished eight cell lines (NZM09, NZM11, NZM19, NZM22, NZM40, NZM52, SK-MEL-28, UACC62), and consisted of down-regulation of genes involved in neural crest and melanocyte development, differentiation, and pigmentation (e.g., *EDNRB, MITF, MLANA, TYR*; Table S1), and up-regulation of genes related to angiogenesis, neurogenesis, immunomodulation, and interaction and remodelling of the extracellular environment (e.g., *HIF1a, NRP1, PLAUR, TGFBI*; Table S1); Motif 2 distinguished the remaining 19 cell lines, and showed a pattern of gene expression that tended to be the inverse of Motif 1, with down-regulation of extracellular remodelling genes and up-regulation of *MITF* and melanocyte lineage markers, although not all Motif 2 cell lines had higher levels of *MITF* expression (e.g. NZM57). SK-MEL-28, a melanoma cell line considered to express relatively low levels of MITF [18], clustered with the lower *MITF* Motif 1 cell lines as expected (Figure 1A). One of the Motif 2 cell lines, NZM04, showed relatively low levels of MITF targets (e.g. *DCT*, *CDK2*, *BCL2*, *GPR143*) despite having relatively higher *MITF* expression (Figure 1B, C), indicating that there are probably melanoma sub-types present within the broad Motif 1 and 2 classifications, and that not all cell lines with lower *MITF* have a Motif 1 expression profile.

### Microarray Validation

Array-based cell line classification was validated by using quantitative real-time PCR (qPCR), western blotting, and immunofluoresence on selected cell lines and genes. Unsupervised clustering of normalised qPCR data separated randomly selected NZM cells into Motif 1 and 2 groups (Figure 1B). Western blots confirmed that in addition to MITF, protein levels of MITF transcriptional targets CDK2, BCL2, and MLANA were also markedly decreased in lower MITF Motif 1 cell lines (NZM09, NZM11, NZM40) compared to Motif 2 (Figure 1C). In accordance with the microarray, qPCR, and western blot data, immunofluoresence suggested differential expression of both MITF and MLANA in representative Motif 1 and 2 NZM cells, with strong nuclear signal for MITF and strong cytoplasmic signal for MLANA in NZM06 compared to NZM09 (Figure 1D).

### Gene Expression Motifs Correlated with Migration Potential *In Vitro*

**Figure 2:**
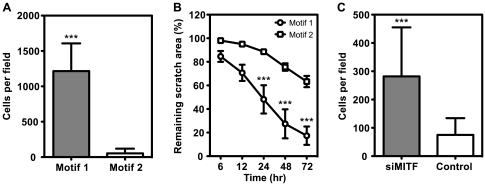
Motif 1 cell lines showed greater motility and migration *in vitro*. (A) The number of Motif 1 cells (NZM09, NZM11, NZM22, NZM40, NZM52) that migrated through pored membranes in transwell (Boyden Chamber) assays was approximately 23-fold more than Motif 2 cell lines (NZM06, NZM12, NZM15, NZM42, NZM45; mean±SD is shown from the combined data of three separate experiments for each cell line; *** p<0.0001, t test). (B) Motif 1 cell lines NZM09 and NZM40 were significantly faster at wound repair in 2D scratch assays than Motif 2 NZM06 and NZM42 cell lines (mean±SEM; n = 3; *** p<0.001, two-way ANOVA). Representative movies of individual scratch assays for NZM09 and NZM42 are provided as supplementary Movie S1 and S2, respectively. (C) siRNA-mediated knockdown of MITF caused an almost 4-fold increase in migration of weakly invasive Motif 2 cell lines (NZM06, NZM15) in transwell assays compared to non-targeting siRNA controls (mean±SD from three separate experiments; *** p<0.0001, t test). MITF knockdown was confirmed by using qPCR and western blot (Figure S2).

We suspected that lower MITF levels in Motif 1 cell lines reflected a de-differentiated cell type with higher migratory ability and therefore invasive potential, and tested this possibility by using scratch and transwell (Boyden chamber) assays as measures of cell motility and migration. Motif 1 cell lines showed a 23-fold higher capacity for migration in transwell assays than Motif 2 cell lines (Figure 2A), and were significantly faster at wound repair in scratch assays than Motif 2 cell lines (Figure 2B; Movie S1, Movie S2). siRNA-mediated MITF knockdown in weakly invasive NZM06 and NZM15 cells caused an average 4-fold increase in migration in transwell assays (Figure 2C; Figure S2), confirming that relative MITF expression was central to the observed difference in invasive potential between Motif 1 and 2 cell lines, and consistent with Motif 1 representing an invasion signature for melanoma cells *in vitro*. In NZM cells, there was no obvious inverse relationship between invasive potential and growth rate, as reported elsewhere [19], with invasive NZM40 cells proliferating significantly faster than either invasive NZM09 cells or weakly invasive NZM06 and NZM42 cells (Figure S3).

### *MITF* Correlation with *CD200*, *BRN2*, and Genomic Copy Number

MITF was reported to be regulated by ERK-activating BRAF mutations [20], so we investigated whether differential *MITF* expression could be explained by using *CD200* as a proxy for ERK activation [21]. *MITF* transcript levels did not correlate with *CD200* expression in NZM cell lines (Figure S3), whereas *MLANA*, a transcriptional target of MITF, strongly correlated with *MITF* expression as expected (Figure S3). Further, expression of the *POU3F2* (*BRN2*) transcription factor, which was reported to be a negative regulator of *MITF* expression [22], did not inversely correlate with *MITF* transcript levels in NZM cell lines (Figure S3). Genomic amplification of the *MITF* locus, which may occur in 10–15% of metastatic melanomas [23], was unlikely to underlie the differential expression of *MITF* between our Motif 1 and 2 NZM cell lines as there was no difference in MITF copy number between NZM cell lines with varying levels of relative *MITF* gene expression (Figure S3).

### The Invasion Signature Classified Independent Cell Line and Tissue Data

**Figure 3:**
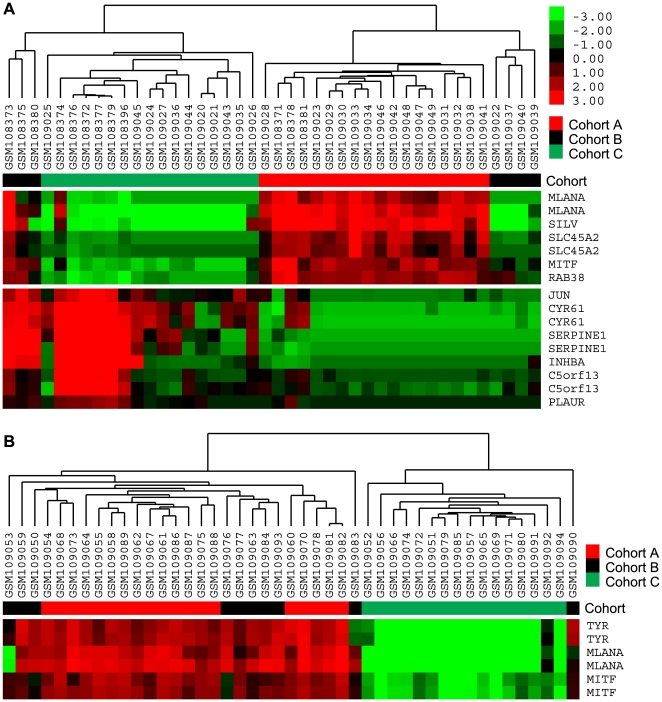
External validation with independent cell line data. Unsupervised clustering by using our 96 gene invasion signature on the combined Zurich and Philadelphia (A), or Mannheim (B) cell line data of Hoek et al. [15] grouped cell lines into the cohorts of differing invasive potential originally identified by those authors, with Motif 1 corresponding to strongly invasive cohort C cell lines, and Motif 2 to weakly invasive cohort A cell lines.

**Figure 4:**
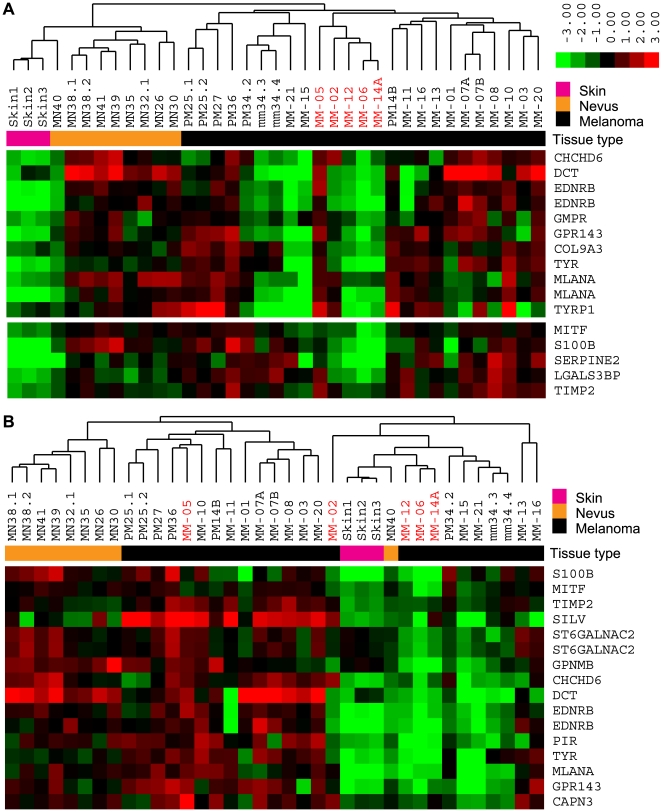
External validation with independent tissue data. (A) Unsupervised analysis by using our filtered gene list (572 transcripts) on the tissue data of Haqq et al. [14] accurately classified skin, nevus, and melanoma tissue. MN, melanocytic nevus; PM, primary melanoma; MM, metastatic melanoma. (B) Application of our 96 gene invasion signature to the tissue data of Haqq et al. identified skin-like and nevus-like tumour samples representing Motif 1- and Motif 2-expressing tumour samples, respectively. Samples in red were derived from patients reported to have “type 1” tumours by the original authors.

Having shown that our 96-gene invasion signature could predict invasive potential *in vitro*, we wished to confirm that our results represented melanoma-specific differential gene expression by performing unsupervised hierarchical clustering on a selection of publicly available data that included both melanoma and non-melanoma tissue samples. Application of our 96-gene invasion signature to unsupervised analysis of the Zurich, Mannheim, and Philadelphia data sets of Hoek et al. [15] could convincingly recapitulate the weakly and strongly metastatic cell line cohorts identified by those investigators, (Motif 1 = cohort C; Motif 2 = cohort A; Figure 3A and B). Conversely, the invasive/proliferative signature reported by Hoek et al. [15] could group our NZM cell lines into the same Motif 1 and 2 groups as our signature (not shown), providing further evidence that a core network of MITF-mediated gene expression contributes to invasive potential in melanoma cells. In the combined Zurich and Philadelphia data, the cohort B cell lines of Hoek et al. [15] flank the Motif 1 and Motif 2 cell lines (Figure 3A) suggesting they represent patterns of gene expression intermediate to Motif 1 and 2. In the tissue data of Haqq and colleagues [14], our 572 gene list could clearly cluster melanocytic nevi and skin away from primary and metastatic melanoma tumour samples (Figure 4A). Our 96-gene invasion signature further separated the samples into “skin-like” and “nevus-like” primary and metastatic tumour samples, which corresponded with Motif 1 or Motif 2 gene expression, respectively (Figure 4B). Four of five patients reported by Haqq et al. as “type 1”, and to have shorter survival (MM-02, MM-06, MM-12, MM-14A), had the more invasive Motif 1 gene expression profile suggesting that less melanocytic tumours with relatively lower levels of MITF might have worse prognosis. Further, the primary tumour of patient MM14 of Haqq et al. had Motif 2 expression whereas a lymph node metastasis from the same patient showed more invasive Motif 1 expression (Figure 4B).

In the tissue data of Riker et al. [16] the invasion signature genes correctly classified non-melanoma from melanoma tissue samples, and grouped primary and metastatic tumours as either Motif 1 and clustering with invasive A375 cell lines [24], or similar to higher MITF Motif 2 cell lines NZM12 and NZM15 (Figure S4). Unsupervised analysis of just the melanoma samples revealed two main clusters consisting of Motif 1 and Motif 1-like tumours, or Motif 2 and Motif 2-like tumours (Figure S4). The Motif 1- and 2-like tumours, like the cohort B cells of Hoek et al., may represent transitional states of gene expression between the extremes of invasive Motif 1 and weakly invasive Motif 2. Notably, there was no clear relationship between expression profile and tumour stage, with both primary and metastatic tumours showing invasive Motif 1.

Our invasion signature could classify skin, benign nevi, and primary melanomas in the data of Talantov et al. [25], and like the Riker and Haqq data, identified nevus-like tumours (Figure S5). When clustering only tumour samples independently of skin and nevi in the Talantov data by using our invasion signature genes, eight primary melanomas exhibited an invasive Motif 1 pattern of gene expression, with 13 showing a Motif 2 profile (Figure S5). The nevus-like tumours showed a Motif 1-like expression profile when compared to just the other tumours (Figure S5), but with increased expression of some pigmentation genes, e.g. *DCT*, and displayed a less marked inverse relationship between lineage specification and extracellular remodelling genes, which suggested an alternate or transitional state of invasion-related gene expression was captured in these samples. Finally, we found high concordance in the relative expression of our invasion signature genes when comparing our data with that generated by Folberg and colleagues in comparing highly invasive with weakly invasive uveal melanoma cell lines [26] (Supplementary Information S1), suggesting that the invasion signature is not specific to cutaneous melanoma.

## Discussion

Metastatic melanoma is a clinically heterogeneous disease that responds unpredictably to treatment, and remains largely refractory to current therapeutic options. We have been generating melanoma cell lines in order to develop new therapies for metastatic melanoma, and to identify markers that might better predict response to therapy, tumour aggression, and patient survival. In this study, our gene expression profiling and knockdown experiments provided evidence that a core network of MITF-mediated transcription formed a significant component of a gene signature that correlated with the invasive potential of metastatic melanoma cell lines *in vitro*. This is consistent with the work of other investigators that showed reduced levels of MITF was associated with increased invasiveness in melanoma cells [27], whereas MITF over-expression suppressed melanoma metastasis in mouse xenograft tumours [28]. Further, our data suggests that acquisition of a more invasive phenotype in melanoma requires both relative down-regulation of developmental and lineage-specific pigmentation genes partnered with up-regulation of genes likely to mediate interaction with the extracellular microenvironment of the tumour.

A number of mechanisms have been postulated to alter MITF expression in melanoma. BRN2 has been reported to be a negative regulator of MITF in melanoma cells [22], yet we were unable to show any correlation between *MITF* and *BRN2* transcription in this study, with any potential correlation tending towards positive rather than negative at the transcript level. We found that *MITF* transcript levels did not correlate with *CD200*, a proxy of ERK activation, which agrees with the conclusions of others that if ERK activation caused by, for example, BRAF mutation, regulates *MITF* expression in melanoma then it is only weakly so at the level of transcription. Thus, multiple factors in addition to MITF may be responsible for the differential expression of developmental genes associated with the invasive potential of metastatic melanoma cells. The regulatory contribution of MITF may be masked by the combined contribution of these other regulators.

Thirty-five of the genes in our 96-gene invasion signature are known or predicted targets of MITF [18], and although experimentally confirmed MITF targets such as *MLANA*, *RAB27A*, and *GPR143* corresponded closely with *MITF* expression in our data, some did not, suggesting a disconnect in MITF signalling for some MITF targets in certain melanomas. For example, MITF was shown to bind and transactivate the *HIF1a* promoter in mouse B16 melanoma cells [29], yet in our study *HIF1a* was inversely correlated with *MITF* expression, particularly in the invasive cell lines, suggesting de-regulated MITF and/or cAMP signalling in NZM cells, or that B16 cells may not be a good model of human metastatic melanoma. The inverse correlation between *MITF* and *HIF1a* was also observed upon application of our signature to independent tumour data suggesting it is a biologically relevant *in vivo*. Investigations into whether MITF transcriptional pathways remain intact in the NZM panel, including whether the putative PAX3/MITF signalling axis is unaltered in melanoma cells, are being reported elsewhere (He et al., submitted).

Recently, Hoek et al. [15] published a 105-gene expression signature able to predict the invasive/proliferative potential of melanoma cells. There is a 24-gene overlap between our 96-gene signature and that of Hoek et al., with 18 of the genes in common, including *MITF*, involved in melanin biosynthesis, pigmentation, development, and lineage specification (Supplementary Information S1), which reinforces the importance of these pathways in melanoma. However, the majority of the genes in our invasion signature are not in common with the Hoek signature, suggesting we have identified novel genes involved with melanoma invasion. The differences between the two invasion signatures could reflect the alternative array platforms, diversity between different melanoma cell lines upon adaptation to growth *in vitro*, or intrinsic tumour heterogeneity.

A number of the differentially expressed transcripts in our invasion signature that are involved in interaction and remodelling of the extracellular environment have previously been associated with increased malignancy or worse prognosis in cancer. The metastasis-associated gene *S100A4* showed increased expression in invasive melanoma cell lines in this study, with *S100A4* over-expression previously associated with a poor prognosis in a variety of human neoplasms such as stomach, colon, breast, melanoma, gallbladder, and pancreatic cancer (reviewed in [30]). S100A4 may mediate invasive potential by regulating matrix metalloproteinases and tissue inhibitors of matrix metalloproteinases such as TIMP2 [31]. TIMP2 is involved in matrix degradation and invasion, and is down-regulated in Motif 1 cell lines in this study, which is consistent with its role in inhibiting matrix metalloproteinases, and agrees with the inverse correlation shown between S100A4 and TIMP2 in osteosarcoma cells [31]. Further, over-expression of TIMP2 in tumour stroma was associated with increased disease-free survival in prostate cancer [32], and down-regulation of MMP2 by TIMP2 over-expression reduced tumour growth and metastatic potential in a rat model of breast cancer-associated brain metastasis [33]. Transcripts of the extracellular matrix protein TGFBI were elevated in our more invasive cell lines. TGFBI has been identified as a member of a metastasis network in oesophageal squamous cell carcinoma [34], with higher expression noted in renal, gastrointestinal, and brain tumours [35], and linked to increased metastatic potential and poorer prognosis in colon cancer [36]. *VCAN* expression is elevated in our invasive Motif 1 NZM cells, which is in agreement with a recent report that VCAN is up-regulated in invasive human melanoma cells via a TCF4/AP1-mediated mechanism [37].

**Figure 5:**
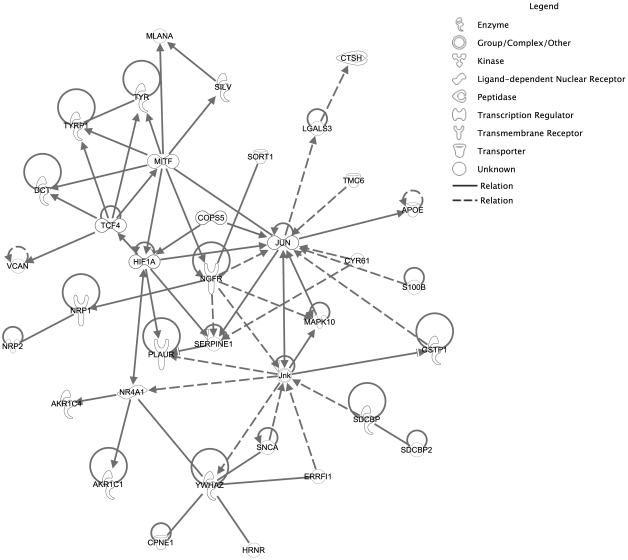
A model linking expression of lineage specification and extracellular sensing genes to invasive potential in melanoma. Pathway analysis suggested that melanoma invasive potential may be mediated by the intersection of MITF-driven transcriptional networks with pathways involved in HIF/JUN activation and response to hypoxia.

Pathway analysis by using our invasion signature genes linked networks that connect hypoxia to invasive potential (Figure 5), which provides additional strength to the suggestion that hypoxia may be a significant driver of phenotype switching in melanoma [19] and contributes to melanoma progression [38]. Hypoxia can promote lymph node metastasis via up-regulation of PLAUR [39], and up-regulation of *HIF1a*, *JUN* and *PLAUR* in our invasive cell lines hints at possible activation of JUN/AP1 and HIF/ARNT pathways in melanoma (Figure 5). The presence of differential signature gene expression in *in vivo* data suggests that relative expression of oxygen-responsive genes is not simply a consequence of the adaptation of NZM tumour cells to culture in our model system, but is likely a snapshot of prevailing transcription at the time the tumour sample was removed.

The identification of skin-like and nevus-like primary and metastatic tumours in the data of Haqq, Riker, and Talantov that corresponded with more or less invasive patterns of gene expression respectively, bolsters our initial suggestion that invasive Motif 1 NZM cells with relative depression of pigmentation gene transcription probably reflected a more de-differentiated and less melanocytic cell type than Motif 2. Further, this also suggests that invasive potential may not evolve solely as a function of melanoma stage and progression, but may exist early in the transformation process and act to prime neoplastic cells for metastatic invasion in response to certain environmental queues, e.g. hypoxia. Conversely, those tumours with a Motif 2 signature gene profile appear to have relatively intact signalling pathways that control pigmentation and differentiation, similar to that operating in normal nevi, and may not be as responsive to changes in the tumour microenvironment, resulting in reduced invasive capacity. However, the fact remains that Motif 2 NZM cell lines, despite being relatively less invasive than Motif 1 cells, were derived from metastatic melanoma samples, and had thus gained an invasive phenotype at some point during tumourigenesis. This supports the notion of phenotype switching proposed by Hoek and colleagues, which suggests invasive potential is a dynamic, non-linear process that transitions from more to less invasive states depending on the microenvironment encountered by the tumour during the course of disease progression.

The ability of our invasion signature to classify tumour and cell line transcription in multiple data sets is compelling evidence that our *in vitro*-derived motifs represent patterns of differential gene expression that may define invasive capacity of melanoma cells *in vivo*. However, although Motif 1 and Motif 2 classification - which probably represent the extremes of the invasive phenotype - can easily and reproducibly be found in publicly available data, the lack of corresponding clinical data that might give clues to the prognosis and aggression of the tumours means that we are as yet unable to confirm whether our invasion signature is a stage-independent indicator of metastatic potential and survival. Nonetheless, this study has identified a number of genes involved in development, lineage specification, and interaction with the tumour microenvironment that may drive invasion, and could be new targets for improved monitoring and treatment of metastatic melanoma, the significance of which we aim to investigate in future studies.

## Materials and Methods

### Cell Lines

The 25 NZM cell lines used for this study were generated from ethically consented and pathologically confirmed metastatic melanoma samples as previously described [40], [41], and grown at 37°C (5% C02) in MEM-alpha media (Invitrogen) supplemented with 5% foetal calf serum (FCS) and 1% insulin-tranferrin-selenium (Roche). UACC62 and SK-MEL-28 were obtained from ATCC and grown in DMEM (Invitrogen) containing 10% FCS and 1% penicillin/streptomycin. NZM01 and NZM02 were derived from different tumours from the same patient at different surgeries. NZM07.2 (p53 mutant) and NZM07.4 (p53 wild type) were subsequently derived from the parental NZM07 cell line.

### *In Vitro* Motility and Migration Assays, siRNA Transfection

For motility assays, randomly chosen Motif 1 (NZM09, NZM40) or Motif 2 (NZM06, NZM42) cells were grown to confluence in normal media in a 6-well plate, scratched with a 200 µL plastic pipette tip, then transferred to a heated-stage assembly on an Olympus IX71 inverted microscope. Scratch closure was captured by time-lapse photography over 3 days. Scratch assays were quantified by generating still images from the time-lapse movie at specific time-points with Imovie (Apple Inc.), and scratch area measured by using a pixel intensity threshold with ImageJ software [42] that allowed exclusion of cells that had migrated into the scratch from the surface area calculation. Representative time-lapse movies of faster-migrating NZM09 cells and slower-migrating NZM42 cells are provided as supplementary Movie S1 (NZM09) and Movie S2 (NZM42).

For migration assays, all adherent Motif 1 NZM cell lines (NZM09, NZM11, NZM22, NZM40, NZM52) and five randomly chosen Motif 2 cell lines (NZM06, NZM12, NZM15, NZM42, NZM45) were used, with 1×10^5^ cells seeded into transwell inserts with 8 µm micropore filters (Becton Dickinson) in 200 µL media. Media containing 10% FCS was added to the lower chamber as chemoattractant. After 24 hours, cells on the upper side of the filter were removed with a cotton swab, with the remaining cells fixed and stained using a standard haematoxylin and eosin protocol, then imaged by using an Olympus IX71 inverted microscope. Five random fields of view were captured per transwell insert, and the number of cells that had migrated to the bottom side of the membrane was counted by using the particle counting module of ImageJ after size and pixel intensity thresholding. Each assay was repeated in three independent experiments resulting in 15 fields of view for each cell line. siRNA-mediated knockdown of MITF was performed in two randomly chosen Motif 2 cell lines (NZM06, NZM15) by using reverse transfection with Lipofectamine RNAiMAX (Invitrogen) and a pre-designed siRNA targeting *MITF* (Ambion) according to the manufacturer's instructions, with a final siRNA concentration of 5 nM. MITF knockdown was confirmed by using qPCR and western blotting. Non-targeting negative control experiments were performed by using an siRNA against the non-mammalian Luciferase gene (GL2) at 5 nM final concentration. Sense-strand siRNA targets sequences were: MITF (Ambion ID#: 3816), 5′-GGACAAUCACAACCUGAUUtt-3′; Luciferase, (Dharmacon), 5′-AACGUACGCGGAAUACUUCGAtt-3′. Cells were exposed to transfection reagents for 24 hours before re-seeding into transwell inserts for migration assays, as described above, with migrated cells measured 48 hours post-transfection.

### Cell Proliferation

Proliferation of cell lines was measured by using an MTT cell proliferation kit (Roche) according to the manufacturer's instructions. Cells (5×10^5^) were seeded in 96-well plates, and incubated for 24, 48, or 72 hours. Ten µL of MTT reagent (5 mg/mL in PBS) was added to each well and incubated for 4 hours at 37°C. The resulting formazan product within the cells was dissolved in 100 µL of 10% SDS in 0.01 M HCl. Optical density (570 nm) was measured by using a PolarStar Optima micro plate reader.

### RNA Isolation and Amplification

Total RNA was isolated from cultured cells by using a combination of Tri Reagent (MRC) and column-based purification (RNeasy, Qiagen; or Purelink, Invitrogen) according to the manufacturer's instructions (a detailed protocol is available at http://openwetware.org/wiki/Eccles:RNA_extraction_AJ). Total RNA was eluted in nuclease-free water, quantified by using a Nanodrop ND-1000 spectrophotometer, then subjected to quality assessment by using an Agilent Bioanalyzer 2100. The Bioanalyzer RNA Integrity Number (RIN) ranged from 8.8–10 for all RNA samples. Antisense RNA (aRNA) was generated from 500 ng total RNA by using an Amino Allyl MessageAmp II amplification kit (Ambion) according to the manufacturer's instructions. For Affymetrix arrays, 200 ng of total RNA was amplified by using a MessageAmp Premiere kit (Ambion) according to the manufacturer's instruction.

### Reverse Transcription and Quantitative PCR

cDNA was generated from 100 ng total RNA by using SuperScript III (Invitrogen) according to the manufacturer's instructions. Transcript abundance was measured by using Platinum SYBR Green qPCR SuperMix-UDG with ROX reference dye (Invitrogen) on an ABI 7300 Real-Time PCR System. qPCR reactions were performed in duplicate with 2.5 ng template cDNA (RNA equivalent) per 20 uL reaction. Cycling conditions were 50°C for 2 min., 95°C for 2 min., then 40 cycles of 95°C for 15 sec./60°C for 1 min., followed by melting curve analysis. For validation of microarray results, comparative qPCR was performed for selected genes on randomly chosen NZM cell lines, with quantification cycle (Cq) values normalised to total RNA input and converted to quantities relative to the cell line with the lowest abundance of a given gene by using the delta-Cq method. qPCR was performed on different RNA samples to those used for microarray analysis. For MITF genomic copy number measurements, DNA was extracted from NZM cells by using a Purelink Genomic DNA Mini Kit (Invitrogen), with 2 ng genomic DNA used as template for qPCR, and qPCR reagents and cycling conditions as described above for transcript measurement. MITF genomic copy number was normalised to LINE1 copy number [43] and expressed relative to a normal human DNA sample by using qBase software and the delta-delta-Cq method. The normal genomic DNA sample was kindly provided by Prof. Stephen Robertson (University of Otago). All primer sequences used for this study are provided in Supplementary Information S1.

### Microarray Hybridisation

Spotted oligonucleotide microarrays were generated at the Otago Genomics Facility (University of Otago) by printing a human 20K oligonucleotide set (MWG Biotech) onto epoxy-coated slides (Schott) using an ESI arrayer. Arrays were blocked immediately before use by pre-hybridisation at 42°C (60 min.) in 1% BSA, 5×SSC, 0.1% SDS. Five ug of Alexa-647-labelled melanoma cell line aRNA was combined with 5 ug Alexa-555-labelled common universal human reference aRNA (Stratagene), fragmented, vacuum concentrated, re-suspended in SlideHyb Glass Array Hybridization Buffer #1 (Ambion), then hybridised to the array under 22 mm ×50 mm LifterSlips (Erie) for 20 hours at 42°C inside a DeRisi-design hybridisation chamber (Monterey Industries). Arrays were washed with 2 x SSC/0.2% SDS (5 min.), 2 x SSC (5 min.), and 0.2 x SSC, dried by centrifugation (200 g, 3 min.), then imaged on an Axon 4000B array scanner. To assess variation in batches of array hybridisations and common reference RNA, replicate hybridisations of RNA from selected cell lines were performed that spanned different hybridisations done at different times with different batches of common reference RNA. RNA for the replicate cell lines was from different biological samples. Two of the cell lines, NZM12 and NZM15, were used for microarray profiling on Affymetrix U133 Plus 2.0 GeneChips, in which 200 ng of Total RNA was amplified by using a MessageAmp Premiere kit (Ambion), with 10-15 ug of biotinylated cRNA used for hybridisation. Affymetrix RNA amplification, hybridisation, washing, and scanning were performed at the Centre for Genomics and Proteomics, University of Auckland.

### Gene Expression Data Analysis

Fluorescent intensity data was extracted by using GenePix Pro 5.0 software, then imported into BRB ArrayTools 3.60 (developed by Dr Richard Simon and Amy Peng Lam, http://linus.nci.nih.gov/BRB-ArrayTools.html) for normalisation (print-tip group lowess smoothing) and data filtering. Genes were excluded if flagged as bad or absent by GenePix, were below an intensity of 150 in both channels, were missing or filtered from more than 50% of the arrays, or did not vary 2-fold or more from the mean value in at least 20% of the arrays (data-filtering outlined in Supplementary Information S1). Class comparison was performed by using the Class Comparison module of BRB ArrayTools. Unsupervised hierarchical clustering of filtered, normalised, and median-centred log2-transformed data was performed by using GenePattern [44] with a Pearson correlation distance measure and an average linkage clustering method, and viewed by using Java TreeView 1.1.1 [45]. After normalisation and filtering, all replicate samples clustered together, including different cell lines from different tumours from the same patient (NZM01, NZM02) and derivative cell lines (NZM07, NZM07.2, NZM07.4), with no scaling required to control for batch-specific biases. The data discussed in this publication have been deposited in NCBI's Gene Expression Omnibus [46] and are accessible through GEO Series accession numbers GSE16249 (Affymetrix data; http://www.ncbi.nlm.nih.gov/geo/query/acc.cgi?acc=GSE16249) and GSE16404 (spotted array data; http://www.ncbi.nlm.nih.gov/geo/query/acc.cgi?acc=GSE16404).

For validation on independent melanoma data sets, Affymetrix array data was imported from GEO into GenePattern as either the original CEL files [15], [16], [47], [48] or as MAS5-normalised SOFT-formatted data files [25]. CEL files were RMA-normalised by using the ExpressionFileCreator module of GenePattern. Expression values for the specific NZM signature genes were extracted by using the SelectFeaturesRows GenePattern module and submitted to hierarchical clustering as log-transformed median-centred data (Pearson correlation, average linkage). The two-colour spotted array data of Haqq et al. was used as provided by the authors in Supplementary Table 10 of their paper [14], and converted to a GenePattern GCT-formatted file for gene extraction and hierarchical clustering (Pearson correlation, average linkage, median-centred). Missing data imputation was performed by using the ImputeMissingValues.KNN module of GenePattern. Gene ontology and pathway construction was performed by using GATHER [49], Cell Illustrator Online v4.0, and Ingenuity Pathway Analysis v12.2, with permutation analysis to estimate false discovery at the pathway level.

### Western Blotting and Immunofluorescence

The steps for protein preparation, electrophoresis and blotting onto nitrocellulose membrane (Amersham) were the same as stated previously [50] except that blotting was carried out using a Western Breeze Immunodetection Kit (Invitrogen). After blocking, the membrane was probed with a primary antibody to either MITF (Zymed; 1∶1000), CDK2 (Santa Cruz; 1∶2000), BCL2 (Santa Cruz; 1∶1000), MLANA (Santa Cruz; 1∶5000). or B-Actin (Sigma; 1∶20000). The membrane was then washed, incubated with secondary antibody, then incubated with chemiluminescence luminol reagents according to the manufacturer's directions. The amplified signals were then detected with Kodak X-OMAT AR film.

For immunofluorescence using cell lines, cells grown to 50% confluency on glass coverslips were rinsed with PBS, and fixed with 4% paraformaldehyde in PBS for 15 min. at room temperature. Coverslips were rinsed with PBS before incubation with 1% BSA in PBS for 30 min. to block non-specific antibody binding, then incubated for 60 min. at room temperature with either mouse monoclonal anti-MLANA antibody, Santa Cruz), or mouse monoclonal anti-MITF antibody (clone C5+D5, Zymed). After rinsing three times with PBS, the coverslips were subsequently incubated with Alexa Fluor 568 goat anti-mouse secondary antibody (Molecular Probes, 1∶2000) in 0.3% BSA in PBS for 30 min. at room temperature in the dark, washed with PBS, and mounted with DAPI (4V,6-diamidino-2-phenylindole)–containing fluorescence mounting solution (Vector Laboratories). Negative control incubations using the same secondary antibody, but omitting the primary antibody were also carried out and showed negative staining. Images were captured by using SPOT v4.6 software and a SPOT RT-SE 6 Slider digital camera (Diagnostic Instruments) connected to a Zeiss Axioplan microscope equipped with an X-Cite 120 fluorescent light source (EXFO).

## Supporting Information

Table S1 The 96 genes identified by class comparison as significantly differentially expressed between higher and lower *MITF* melanoma cell lines, ranked by log2 ratio.
(0.04 MB XLS)

Supplementary Information S1 Data filtering; filtered gene list; class comparison; comparison with Hoek et al. signature; comparison with Folberg et al. data; primers used for qPCR.
(0.67 MB XLS)

Figure S1 Unsupervised hierarchical clustering of metastatic melanoma cell lines by using the filtered list of 572 genes.
(0.54 MB PDF)

Figure S2 siRNA-mediated MITF knockdown in weakly invasive NZM cells.
(0.15 MB PDF)

Figure S3 Proliferation; *MITF* correlation with *BRN2*, *CD200*, and *MLANA*; and *MITF* genomic copy number.
(0.12 MB PDF)

Figure S4 Unsupervised clustering of invasion signature genes in the tissue data of Riker et al.
(0.75 MB PDF)

Figure S5 Unsupervised clustering of skin, benign nevi, and primary melanoma data from Talantov et al.
(0.40 MB PDF)

Movie S1 Time-lapse movie of a scratch assay for NZM09, a fast-migrating Motif 1 cell line.
(1.63 MB MOV)

Movie S2 Time-lapse movie of a scratch assay for NZM42, a slow-migrating Motif 2 cell line.
(1.64 MB MOV)
