# Supplementary material for: A Gene Expression Signature of Invasive Potential in Metastatic Melanoma Cells
Source: PLoS One. 2009 Dec 24;4(12):e8461. doi: 10.1371/journal.pone.0008461 (PMC2794539; doi:10.1371/journal.pone.0008461)
Supplement: Figure S1 — Unsupervised hierarchical clustering of metastatic melanoma cell lines by using the filtered list of 572 genes. (0.54 MB PDF) [file pone.0008461.s003.pdf]

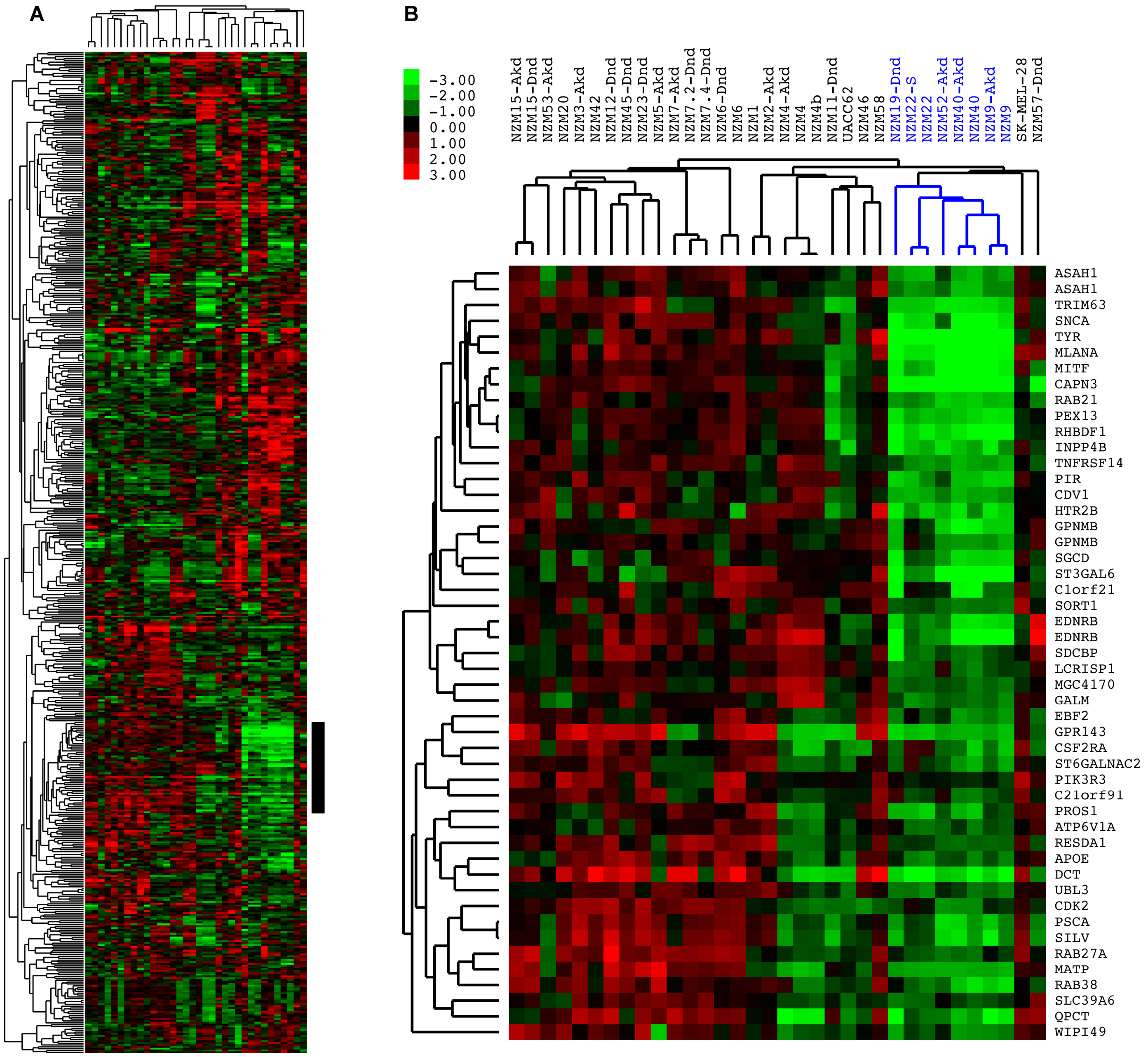

Figure S1. **Unsupervised hierarchical clustering of metastatic melanoma cell lines by using the filtered list of 572 genes.** (A) Two major clusters of cell lines were identified, with a group of cell lines in the right cluster defined in part by differential expression of *MITF* and down stream target genes (black bar). (B) The gene cluster identified by the black bar in (A), with the lower *MITF* group labelled blue. NZM11, UACC62, and NZM57, also located in the main cluster on the right, also showed lower expression of *MITF*.
