# Supplementary material for: A Gene Expression Signature of Invasive Potential in Metastatic Melanoma Cells
Source: PLoS One. 2009 Dec 24;4(12):e8461. doi: 10.1371/journal.pone.0008461 (PMC2794539; doi:10.1371/journal.pone.0008461)
Supplement: Figure S2 — siRNA-mediated MITF knockdown in weakly invasive NZM cells. (0.15 MB PDF) [file pone.0008461.s004.pdf]

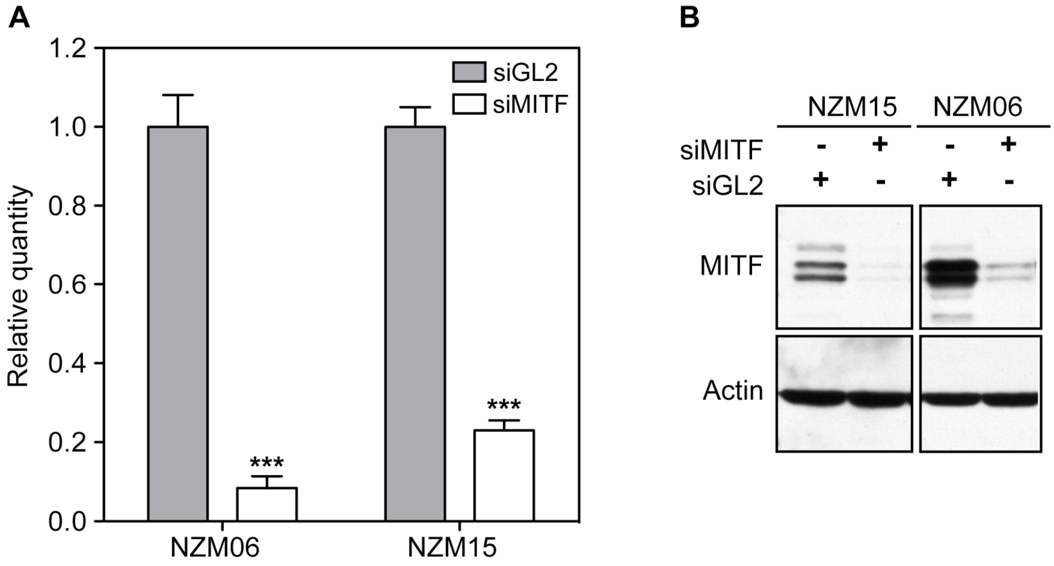

Figure S2. siRNA-mediated MITF knockdown in weakly invasive NZM cells. (A) Treatment of cells with siRNA targeting *MITF* (siMITF) caused a 92% and 77% reduction in *MITF* transcript levels 24 hours post-transfection in NZM06 and NZM15 cells, respectively, relative to non-targeting controls (siGL2) as determined by q-RT-PCR. *MITF* levels are shown normalised to the expression of *RPL32* and *GNB2L1* reference genes and relative to siGL2. Reference gene stability was assessed by genorm software, with M values of 0.042 and 0.084 for NZM06 and NZM15, respectively (n =3; *** $p < 0.001$, t test). (B) Western blots showed a significant reduction in MITF protein levels in siMITF-treated cells compared to siGL2 non-targeting controls 48 hours post-transfection.
