# Supplementary material for: A Gene Expression Signature of Invasive Potential in Metastatic Melanoma Cells
Source: PLoS One. 2009 Dec 24;4(12):e8461. doi: 10.1371/journal.pone.0008461 (PMC2794539; doi:10.1371/journal.pone.0008461)
Supplement: Figure S3 — Proliferation; MITF correlation with BRN2, CD200, and MLANA; and MITF genomic copy number. (0.12 MB PDF) [file pone.0008461.s005.pdf]

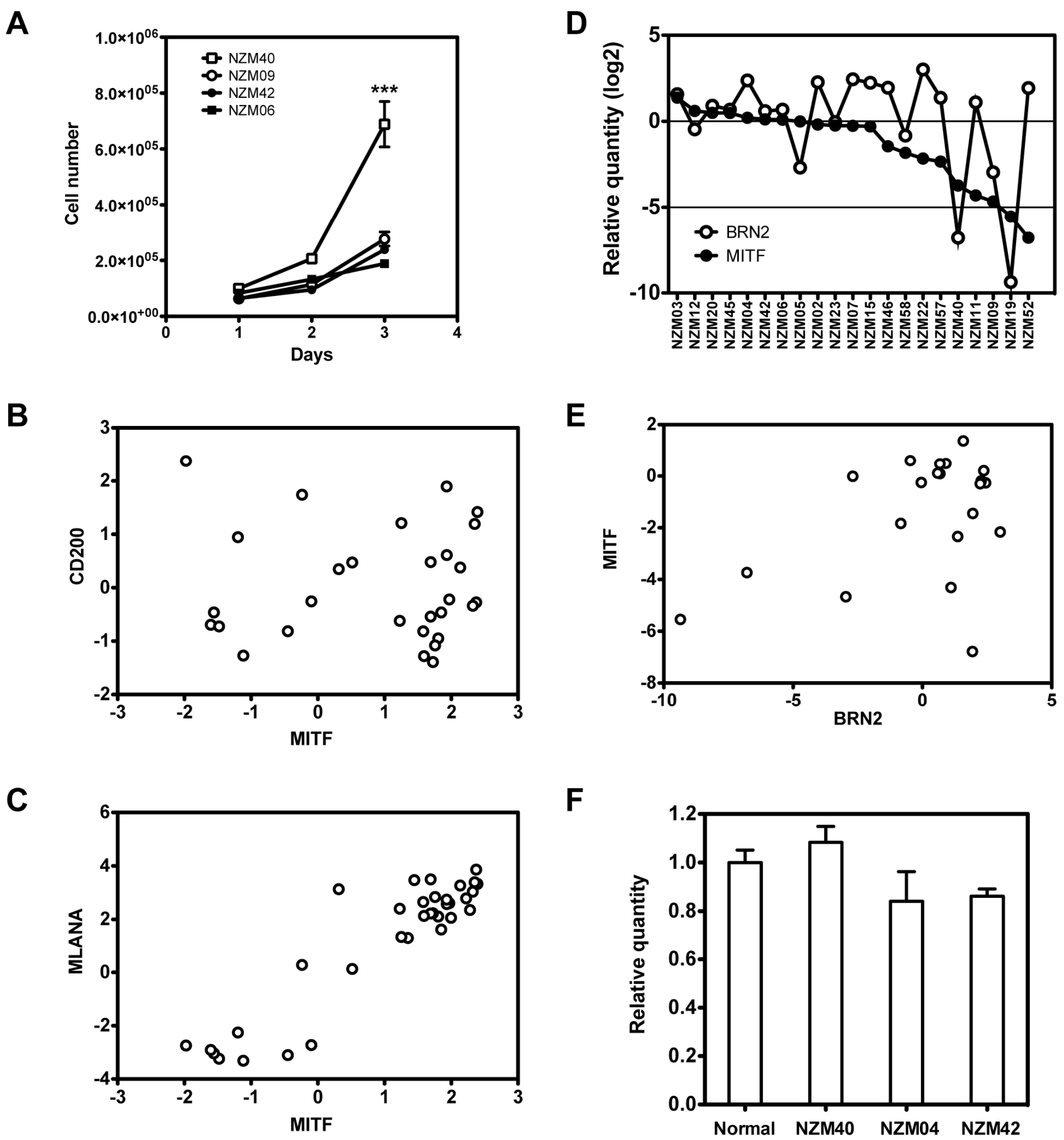

Figure S3. **Proliferation, *MITF* correlation with *BRN2*, *CD200* ,and *MLANA*, and *MITF* genomic copy number.** (A) Proliferation rates of selected Motif 1 and Motif 2 cell lines. Invasive Motif 1 cell line NZM40 proliferated significantly faster than invasive NZM09 cells, or weakly invasive NZM06 and NZM42 cells (mean +/- SEM, n = 3, *** p < 0.001, two-way ANOVA). (B) Expression of *CD200*, a marker of ERK activation, correlated poorly with *MITF* expression (r^2 = 0.00002, p = 0.99). (C) Expression of *MLANA*, a transcriptional target of MITF, correlated strongly with *MITF* expression (r^2 = 0.85, p < 0.0001). The values reported are normalised log2 ratios from the two colour array data. (D) *MITF* and *BRN2* expression did not show an inverse relationship in NZM cells. Samples sorted from highest to lowest *MITF* expression as determined by qPCR, with relative quantities expressed as mean-centred log-transformed delta-Cq values. (E) *MITF* and *BRN2* showed a weak positive correlation in NZM cells (r^2= 0.2, p = 0.04). (F) MITF genomic copy number was not significantly different between DNA from normal human peripheral blood and NZM cells lines with a 16-fold difference in *MITF* transcript abundance. qPCR was normalised to LINE element copy number and is expressed relative to normal DNA.
