# Supplementary material for: A Gene Expression Signature of Invasive Potential in Metastatic Melanoma Cells
Source: PLoS One. 2009 Dec 24;4(12):e8461. doi: 10.1371/journal.pone.0008461 (PMC2794539; doi:10.1371/journal.pone.0008461)
Supplement: Figure S4 — Unsupervised clustering of invasion signature genes in the tissue data of Riker et al. (0.75 MB PDF) [file pone.0008461.s006.pdf]

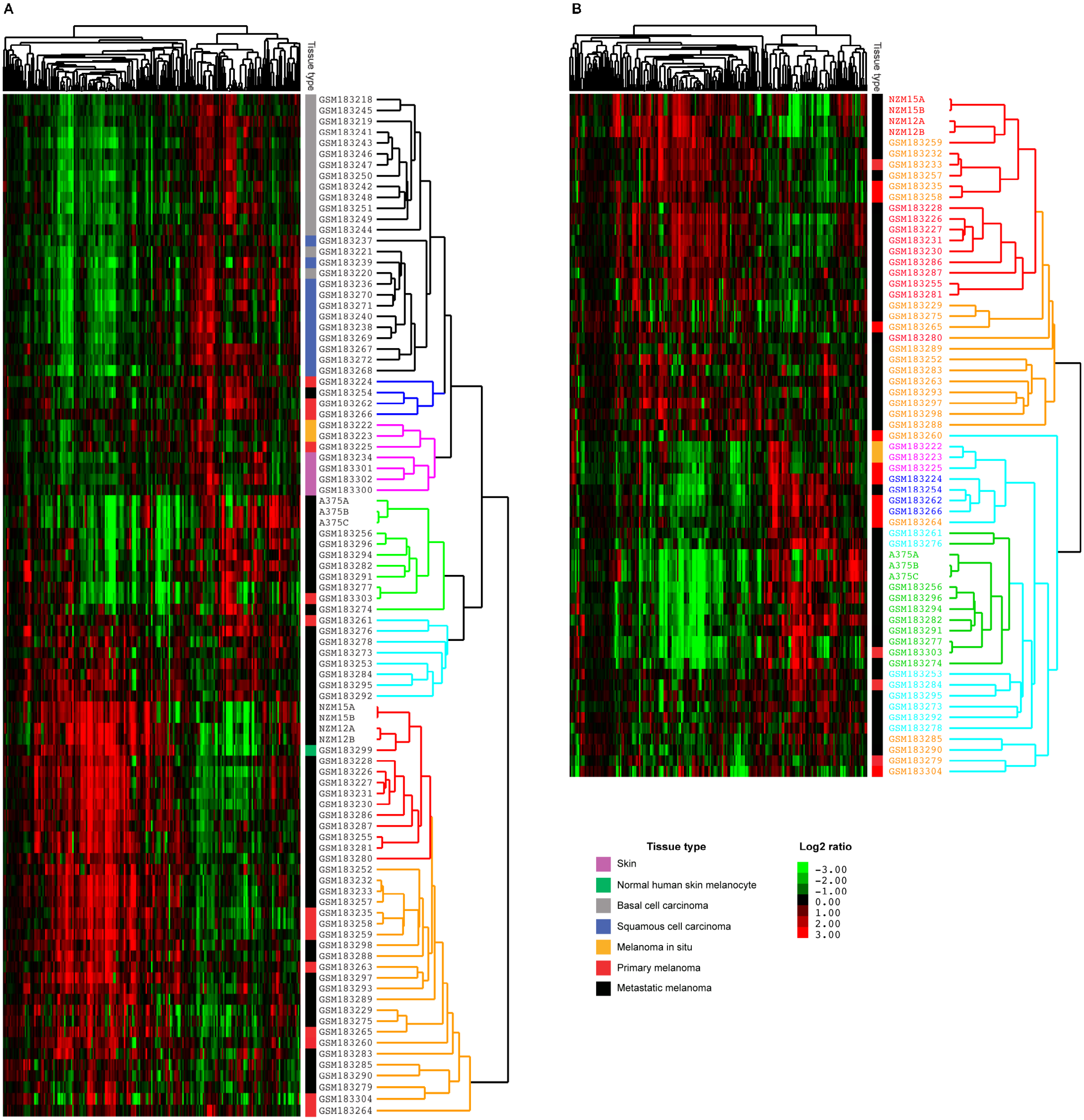

Figure S4. Unsupervised clustering of invasion signature genes in the tissue data of Riker et al. [16]. The 96-gene signature was applied to the melanoma tumour data from Riker et al. combined with publicly available data from strongly invasive A375 cells (Motif 1) [47, 48], and weakly invasive NZM12 and NZM15 cells (Motif 2). (A) non-melanoma skin cancer samples clustered separately from melanoma samples and skin, with invasive Motif 1 samples clustering separately from less invasive Motif 2 samples. Dendrogram colours: black, non-melanoma tumours; blue, basal-like melanoma; pink, skin-like melanoma; green, Motif 1 melanoma; cyan, Motif 1-like melanoma; red, Motif 2 melanoma; orange, Motif 2-like melanoma. (B) Melanoma samples, alone, showed two major clusters consisting of Motif 1 (green) and Motif 1-like tumours (cyan), or Motif 2 (red) and Motif 2-like tumours (orange). Sample name colours correspond with the dendrogram colours in (A). A375A, GSM206443; A375B, GSM217874; A375C, GSM217875.
