# Supplementary material for: A Gene Expression Signature of Invasive Potential in Metastatic Melanoma Cells
Source: PLoS One. 2009 Dec 24;4(12):e8461. doi: 10.1371/journal.pone.0008461 (PMC2794539; doi:10.1371/journal.pone.0008461)
Supplement: Figure S5 — Unsupervised clustering of skin, benign nevi, and primary melanoma data from Talantov et al. (0.40 MB PDF) [file pone.0008461.s007.pdf]

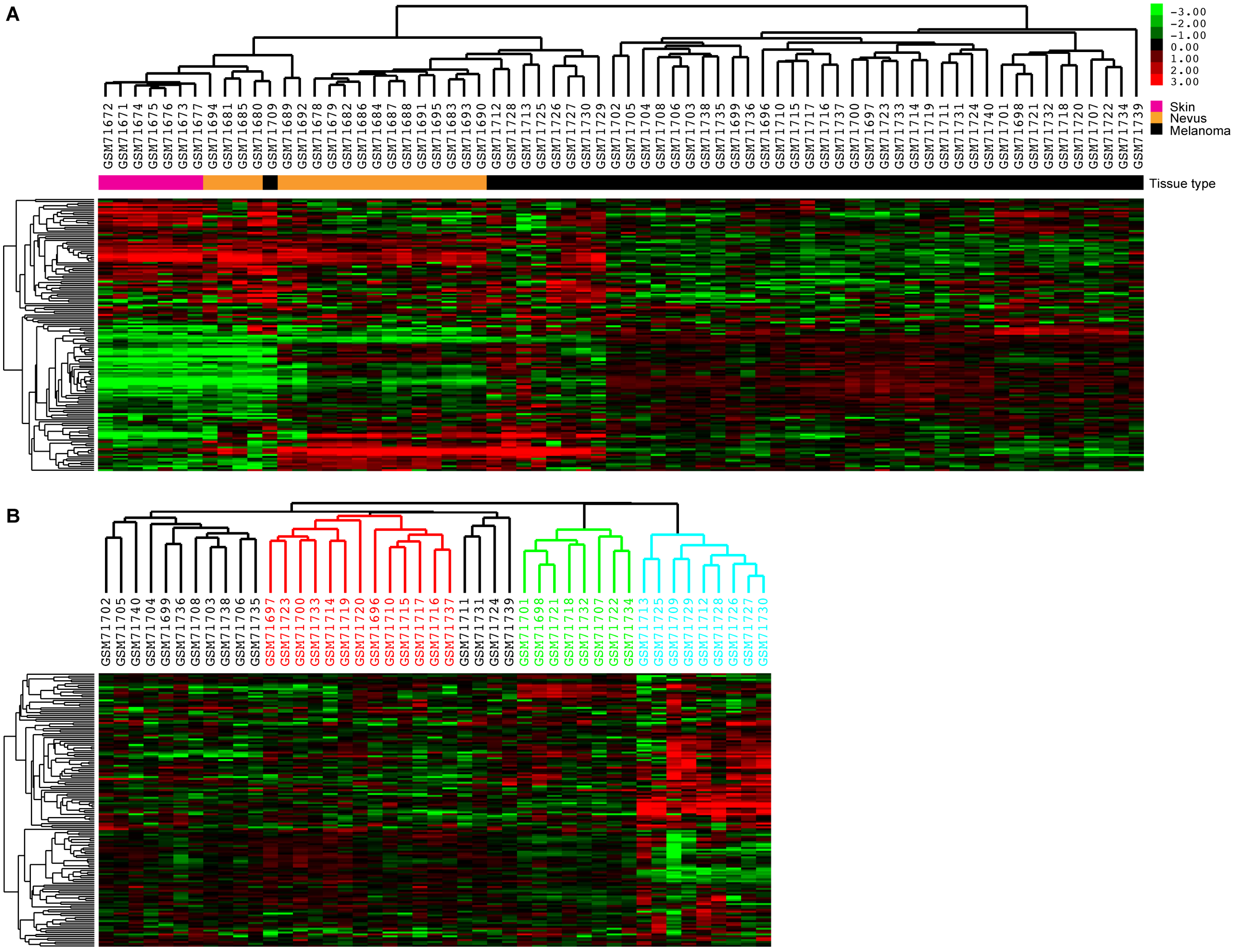

Figure S5. **Unsupervised clustering of skin, benign nevi, and primary melanoma data from Talantov et al.** [25]. (A) In addition to a large cluster of 35 primary tumours (right cluster), the 96-gene invasion signature clustered a single primary melanoma with skin and skin-like nevi (left cluster), and showed eight nevus-like melanocytic primary melanomas (middle cluster). (B) Unsupervised analysis of only the Talantov primary tumour data with the 96-gene invasion signature identified two main clusters consisting of samples with Motif 1 (green) and Motif 1-like (cyan) expression on the right, or Motif 2 (red) and Motif 2-like (black) expression profiles on the left. Interestingly, the Motif 1-like samples (cyan) consist of the skin and nevus-like tumours in (A), which may reflect tumours in a state of transitional gene expression between Motif 1 and 2. For example, although the Motif 1-like samples (cyan) tended to have lower expression of *MITF*, *RAB27A*, *EDNRB*, and *GPR143* similar to Motif 1 samples (green), they also showed higher expression of other developmental genes including *DCT* and *CAPN3* compared to the Motif 1 samples (green), and were similar to Motif 2 for these transcripts.
